# Roles and functions of SARS-CoV-2 proteins in host immune evasion

**DOI:** 10.3389/fimmu.2022.940756

**Published:** 2022-08-08

**Authors:** Farooq Rashid, Zhixun Xie, Muhammad Suleman, Abdullah Shah, Suliman Khan, Sisi Luo

**Affiliations:** ^1^ Division of Infectious Diseases, Chongqing Public Health Medical Center, Chongqing, China; ^2^ Department of Biotechnology, Guangxi Veterinary Research Institute, Nanning, China; ^3^ Guangxi Key Laboratory of Veterinary Biotechnology, Nanning, China; ^4^ Center for Biotechnology and Microbiology, University of Swat, Swat, Pakistan; ^5^ Department of Biotechnology, Shaheed Benazir Bhutto University, Sheringal, Pakistan; ^6^ Department of Medical Lab Technology, The University of Haripur, Haripur, Pakistan

**Keywords:** SARS-CoV-2, immune evasion, structural proteins, non-structural proteins, accessory proteins

## Abstract

Severe acute respiratory syndrome coronavirus 2 (SARS-CoV-2) evades the host immune system through a variety of regulatory mechanisms. The genome of SARS-CoV-2 encodes 16 non-structural proteins (NSPs), four structural proteins, and nine accessory proteins that play indispensable roles to suppress the production and signaling of type I and III interferons (IFNs). In this review, we discussed the functions and the underlying mechanisms of different proteins of SARS-CoV-2 that evade the host immune system by suppressing the IFN-β production and TANK-binding kinase 1 (TBK1)/interferon regulatory factor 3 (IRF3)/signal transducer and activator of transcription (STAT)1 and STAT2 phosphorylation. We also described different viral proteins inhibiting the nuclear translocation of IRF3, nuclear factor-κB (NF-κB), and STATs. To date, the following proteins of SARS-CoV-2 including NSP1, NSP6, NSP8, NSP12, NSP13, NSP14, NSP15, open reading frame (ORF)3a, ORF6, ORF8, ORF9b, ORF10, and Membrane (M) protein have been well studied. However, the detailed mechanisms of immune evasion by NSP5, ORF3b, ORF9c, and Nucleocapsid (N) proteins are not well elucidated. Additionally, we also elaborated the perspectives of SARS-CoV-2 proteins.

## Introduction

Coronaviruses (CoVs) are a diverse family of enveloped positive-sense single-stranded RNA viruses (1–3), infecting humans, avian species, and livestock animals, posing a serious threat to public health and economy (4). CoVs are classified under the order Nidovirales, family Coronaviridae, and subfamily Orthocoronavirinae. The subfamily Orthocoronavirinae is further divided into four genera, i.e., alphacoronavirus (α-CoV), betacoronavirus (β-CoV), gammacoronavirus (γ-CoV), and deltacoronavirus (δ-CoV). The α- and β-CoVs infect only mammals, while the γ- and δ-CoV have a broader host range including avian species (4). Severe acute respiratory syndrome coronavirus 2 (SARS-CoV-2) has been placed in the subgenus Sarbecovirus under the genus β-CoVs (4, 5). Human CoVs, such as HCoV-229E (α-CoV) and HCoV-OC43 (β-CoV), HCoV- NL63 (α-CoV) and HCoV- HKU1 (β-CoV), usually cause mild respiratory tract infections associated with symptoms of the “common cold”. In contrast, SARS-CoV-2, Middle East respiratory syndrome coronavirus (MERS-CoV), and SARS-CoV have been recognized as highly pathogenic (4).

**Figure 1:**
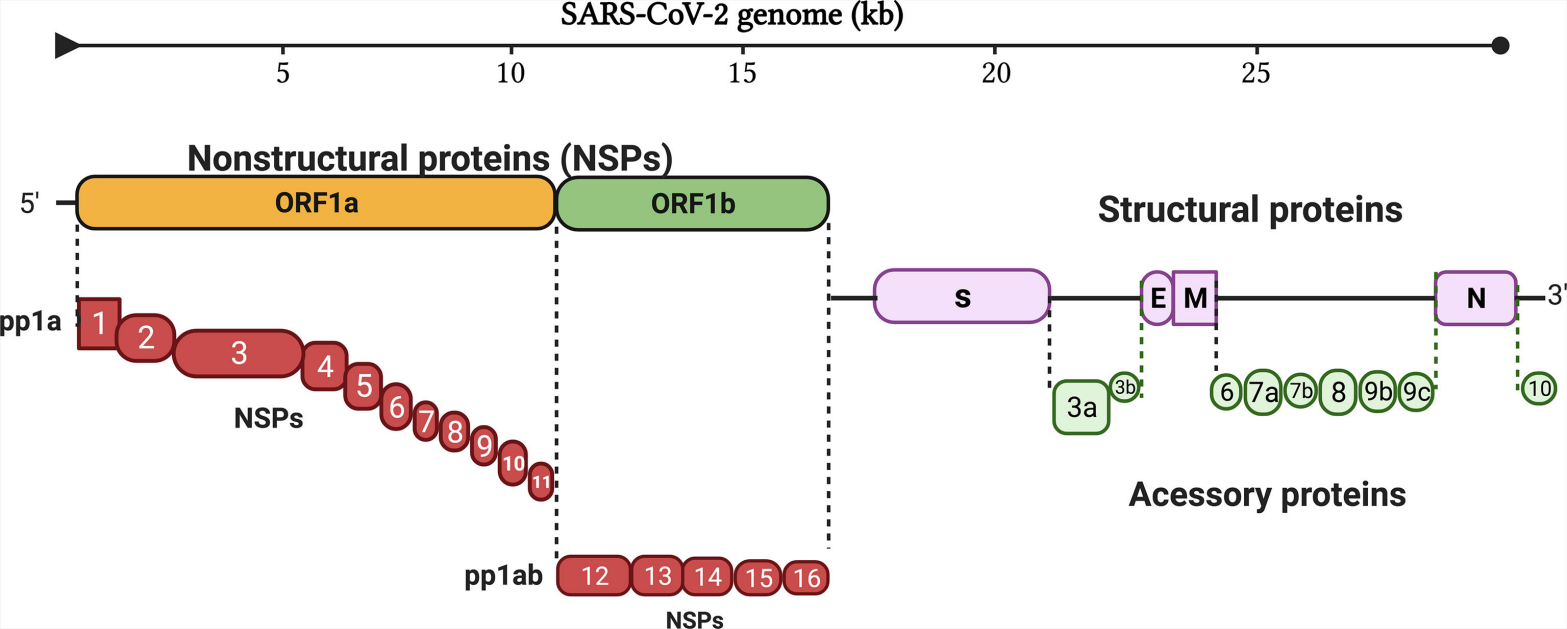
Genome organization of SARS-CoV-2. The genome of SARS-CoV-2 consists of 16 non-structural proteins, ranging from NSP1 to NSP16, four structural proteins (S, E, M and N), and interspersed between these structural proteins are nine accessory proteins. (S, Spike protein; E, Envelop protein; M, Membrane protein; N, Nucleocapsid protein).

The genome of SARS-CoV-2 consists of about 30,000 bases (6, 7) (**Figure 1**). It contains a 5’ cap structure and a 3’ poly-A tail. It has a 5’ open reading frame (ORF) and a 3’ ORF that comprises 2/3 and 1/3 of the complete genome, respectively. After entering the host cell, RNA-dependent RNA polymerase (RdRp) replicates and transcribes the SARS-CoV-2 genome (8). The 5’ ORF (ORF1a/b) is translated into pp1a and pp1ab proteins in the rough endoplasmic reticulum (rER) of the host cell (9). Proteases cleave these proteins and produce 16 NSPs, ranging from NSP1 to NSP16. The 3’ ORF of SARS-CoV-2 has both structural and accessory proteins. There are four structural proteins, i.e., Spike (S), Envelop (E), Nucleocapsid (N), and Membrane (M) proteins (**Figure 1**). The structural proteins assemble and help in the budding of new virions at the ER to Golgi compartment that are suggested to exit the infected cells by exocytosis. S protein recognizes and binds to the receptor, angiotensin-converting enzyme 2 (ACE2) of the host cell, mediating the penetration of the virus into the host cell (7, 10). N protein is multifunctional; its main function is to assemble genomic RNA of the virus into a ribonucleoprotein complex and regulate viral replication (9). E protein regulates the replication, pathogenicity, and virus dissemination (11, 12), while M protein is responsible for the assembly of viral particles (13, 14). Interspersed between these structural proteins are nine accessory proteins, i.e., ORF3a, ORF3b, ORF6, ORF7a, ORF7b, ORF8, ORF9b, ORF9c, and ORF10 (4) (**Figure 1**). The accessory proteins show high variability among CoVs; however, they are conserved within respective viral species to some extent. The accessory proteins do not play roles in virus replication, but they do have important roles in host immune evasion (4).

**Figure 2:**
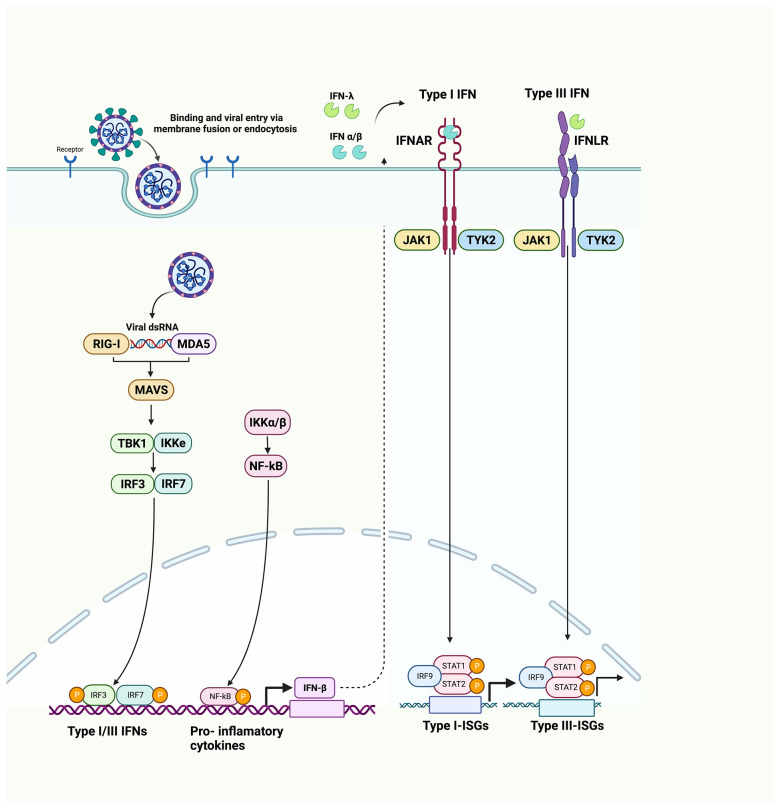
Innate immune system recognition, IFN signaling, and immune evasion by viruses. Upon sensing of viruses by RIG-I and MDA5, NF-κB, IRF3, and IRF7 stimulate the production of proinflammatory cytokines, IFN-I and IFN-III. IFNs are secreted in an autocrine and paracrine manner to induce the expression of ISGs via the JAK/STAT signaling pathway. (RIG-1, retinoic acid-inducible gene I; MDA5, melanoma differentiation-associated gene 5; NF-κB, nuclear factor-κB; IRF3, interferon regulatory factor 3; IRF7, interferon regulatory factor 7).

Interferons (IFNs) are the first line of defense in hosts against invading viruses (15). Innate viral recognition triggers a signaling cascade leading to both nuclear factor-κB (NF-κB)-mediated induction of pro-inflammatory cytokines [e.g., interleukin (IL)-1, IL-6, tumor necrosis factor-α (TNF-α)] and interferon regulatory factor (IRF)3- and IRF7)-mediated induction of type I and type III IFNs (IFN-I and IFN-III) (16). After this, IFN-I and IFN-III responses are activated (17); IFN-I includes IFN-α, IFN-β, IFN-ϵ, IFN-κ, and IFN-ω, while IFN-III is IFN-λ in humans. IFN-I binds to type I IFN receptor (IFNAR) that is ubiquitously expressed in autocrine and paracrine cells. As a result, hundreds of interferon-stimulated genes (ISGs) are activated, which interfere with every step of viral replication. IFN-III binds to IFN-III receptors (IFNLRs), preferentially expressed on myeloid and epithelial cells, thereby producing ISGs (18). IFN-I is a key element in providing efficient protection against viral infections including SARS-CoV-2 (19). IFN-I is produced soon after recognition of pathogen-associated molecular patterns (PAMPs), such as viral mRNA (20). PAMPs are recognized by retinoic acid-inducible gene 1 [RIG-I/DExD/H-box helicase 58 (DDX58)] and melanoma differentiation-associated gene 5 [MDA5/IFN induced with helicase C domain 1 (IFIH1)] (21, 22). Once activated, the RIG-I and MDA5 interact with the caspase activation and recruitment domain (CARD) domain of mitochondrial antiviral signaling protein (MAVS). The activated MAVS recruits several downstream signaling components to the mitochondria. As a result, an inhibitor of NF-κB kinase ϵ (IKKϵ) and TANK-binding kinase 1 (TBK1) are activated that further results in the phosphorylation of IRF3 and IRF7. The phosphorylated IRF3 and IRF7 are dimerized and translocated to the nucleus, where they induce the expression of IFN-I and a subset of ISGs (early ISGs) (23). The secreted IFN-I expression leads to tyrosine kinase 2 (Tyk2) and Janus kinase 1 (JAK1) activation. After activation, STAT1 and STAT2 are phosphorylated (24, 25). The phosphorylated STATs form a heterodimer and associate with IRF9, a DNA-binding protein, to form IFN-stimulated growth factor 3 (ISGF3). The ISGF3 complex translocates to the nucleus and binds with interferon-stimulated response elements (ISREs) at ISG promoters and transcribes its downstream genes (**Figure 2**).

Therefore, hundreds of ISG products are expressed at viral infection sites, producing the antiviral state (25). The ISGs and pro-inflammatory cytokines have diverse functions including inhibition of viral replication, activation, and recruitment of immune cells. IFN-I production is therefore required against viral infections to trigger adaptive immune responses for a longer duration (26, 27).

The transcriptome profiles of different cell types delineate the infection of SARS-CoV-2. The infection triggers a low level of IFN-I and IFN-III, and hence, limited ISG response is produced. However, it does induce pro-inflammatory cytokines (28, 29). Low levels of IFN-I in the serum of coronavirus disease 2019 (COVID-19) patients were detected in the early stages of infection and elevated levels during the advanced stage of infection (29, 30). Thus, ISG induction requires limited IFN-I production, or IFN-I may be produced in specific immune cells. Compared to SARS-CoV, SARS-CoV-2 induces less IFN-I (30). IFN-I deficiency in the blood is an indication of -19 severity, which should be taken seriously (31). SARS-CoV-2 infection also induces the activation of TLR3 and Toll-like receptor 7 (TLR7) RNA sensor pathways in the Clau-3/Medical research council cell strain (MRC)-5 multicellular spheroids (MTCSs) (32). TLR3 acts *via* IRF3 producing IL-1α, IL-1β, IL-4, IL-6, IFN-α, and IFN-β. TLR3 also activates the NF-κB transduction pathway. TLR7 acts *via* NF-κB pathway, inducing IFN-I, IFN-γ, and IFN-λ3. Therefore, TLRs could also be potential targets in controlling the SARS-CoV-2 infection (32). To have a successful infection, SARS-CoV-2 has evolved several strategies to overcome the host immune system. Since its discovery, SARS-CoV-2 has gained significant genetic diversity in all of its genes that is continuously changing the immune capabilities of its host (33). To date, several SARS-CoV-2 proteins are known to have helped in immune evasion. In the following sections, we have discussed the host immune evasion by SARS-CoV-2 proteins and the mechanisms through which they help the virus evade the immune system.

## NSP1 antagonizes IFN-I signaling by inhibiting STAT1 phosphorylation

Previous work on SARS-CoV has reported several roles for NSP1 (34, 35). It suppresses the translation of host proteins by interacting with the 40S subunit and inhibiting the 80S subunit formation (35). It also induces endo-nucleolytic cleavage and subsequently degrades host mRNAs, leading to an inhibition of innate immune responses of host cells (34, 35).

The NSP1 of SARS-CoV-2 and SARS-CoV has 84% sequence identity in amino acid residues. Such high conservation suggests similar biological functions and properties (35). The NSP1 of SARS-CoV contains Lys164 (K164) and His165 (H165) dipeptide motif, which is indispensable for human 40S ribosomal subunit interaction, leading to inhibition of host translation. K164A and H165A substitutions abolish the binding of NSP1 to 40S ribosomal subunit (36). This dipeptide motif is conserved in SARS-CoV-2 NSP1, and a similar function of protein translation inhibition was observed (37, 38). When cells were overexpressed with NSP1 followed by Sendai virus (SeV) stimulation, which is an excellent inducer of RIG-I, the endogenous protein levels of IFN-β, IFN-λ, and IL-8 were significantly reduced. However, the transcription of the corresponding genes was induced by NSP1 overexpression (39). The NSP1 mutant had no inhibitory effects on the protein levels of IFN-β, IFN-λ, and IL-8. Similarly, NSP1 suppressed the luciferase activity driven by ISRE, which is the promoter part of the ISGs (39, 40). However, autophagy was hardly affected by NSP1 expression, even when induced by rapamycin (38, 41). These results delineated that NSP1 almost fully inhibits the translation of IFNs, pro-inflammatory cytokines, and ISGs. All of the above studies suggested that NSP1 of SARS-CoV-2 is one of the main immune evasion proteins (35–38, 40, 41). NSP1, therefore, may be an attractive therapeutic target against COVID-19, but further investigation is required to determine whether NSP1 is the best option for vaccine development (42).

**Figure 3:**
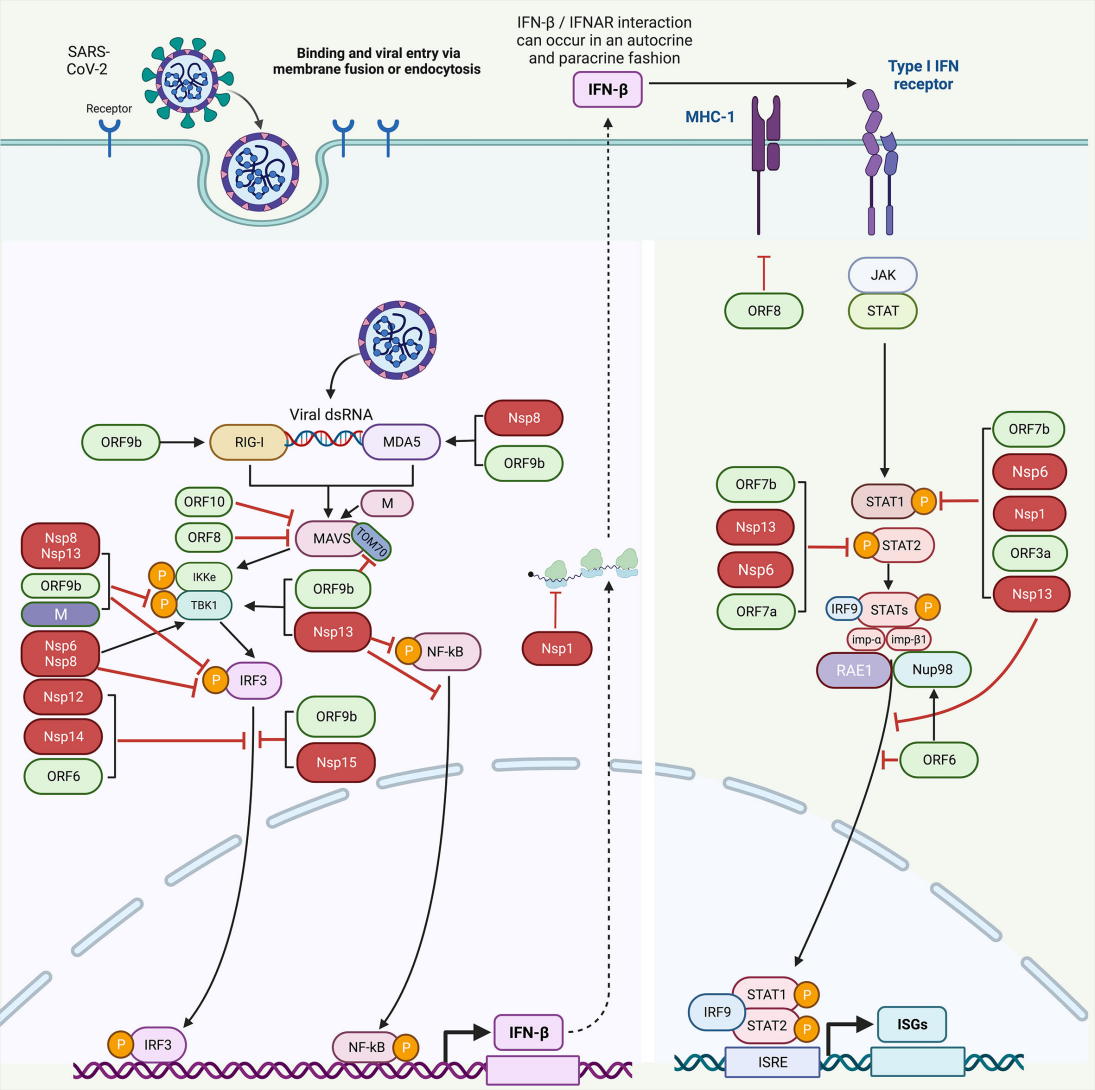
Host immune evasion by SARS-CoV-2 proteins. SARS-CoV-2 triggers IFN signaling pathway after being recognized by RIG-1 and MDA5. Different SARS-CoV-2 proteins interfere with these pathways in different ways. NSP8 and NSP13 inhibit TBK1 phosphorylation. NSP6, NSP8, NSP13, OR9b, and M inhibit IRF3 phosphorylation. NSP12, NSP14, NSP15, ORF6, and ORF9b inhibit the nuclear translocation of IRF3. NSP1, NSP6, NSP13, ORF3a, and ORF7b inhibit STAT1 phosphorylation. ORF9b inhibits TOM70. NSP6, NSP13, ORF7a, and ORF7b inhibit STAT2 phosphorylation. NSP13 and ORF6 inhibit the nuclear translocation of STAT1 to antagonize IFN signaling. ORF6 blocks STAT1 nuclear translocation by interacting with the Nup98-RAE1 complex and disrupts the interaction between Nup98 and importin-β1/importin-α1/PY-STAT1 complex, thus preventing the docking of this complex at the nuclear pore. ORF8 inhibits MHC-I to impair antigen-presenting cells. [NSP, non-structural protein; ORF, open reading frame; TOM70, translocase of outer membrane 70 KDa Subunit; IFN, interferon; STAT, signal transducer and activator of transcription; IRF3, interferon regulatory factor 3; IRF9, interferon regulatory factor 9; NF-κB, nuclear factor-κB; ISGs, interferon-stimulated genes; MHC-I, major histocompatibility complex I; Nup98-RAE1 complex, nucleoporin 98 (Nup98)–ribonucleic acid export 1 (RAE1)].

Another research also determined that NSP1 can inhibit IFN-β production and suppress 98% IFN-β promoter activity *via* the proteins that are both upstream and downstream of IRF3 (43). NSP1 significantly inhibits STAT1 phosphorylation, while STAT2 phosphorylation could be marginally inhibited. Moreover, NSP1 also suppresses the nuclear translocation of STAT1, suggesting the role of NSP1 in inhibiting IFN-I signaling (**Figure 3**, **Table 1**) (43).

**Table 1:** SARS-CoV-2 proteins interfering with IFN induction and signaling.

Protein	Mechanism	Experimental approach	Cellular model	References
**IFN production inhibition**				
**NSP1**	Multiple targets, may be upstream and downstream of IRF3	Luciferase assay	HEK293T cells	(43)
**NSP6**	Suppress IRF3 phosphorylation	Western blotting	HEK293T cells	(43)
**NSP8**	Suppress the phosphorylation of IRF3, TBK1	Western blotting	HEK293T cells	(44)
**NSP12**	Inhibit nuclear translocation of IRF3	Immunofluorescence assays	HEK293T cells	(45)
**NSP13**	Physical binds with TBK1, Suppress the phosphorylation of IRF3, TBK1, and NF-κB, Suppress nuclear translocation of NF-κB	Luciferase assay, Western blotting, Immunofluorescence assays	HEK293T, HeLa cells	(43, 46)
**NSP14**	Inhibit nuclear translocation of IRF3	Luciferase assay, Immunofluorescence assays	293 FT cells	(47)
**NSP15**	Inhibit nuclear translocation of IRF3	Luciferase assay, Immunofluorescence assays	293 FT cells	(47)
**ORF6**	Inhibit nuclear translocation of IRF3	Luciferase assay, Western blotting, Immunofluorescence assays	HEK293T	(43, 48–50)
**ORF9b**	Interact with MDA5, MAVS, TRIF, TBK1, STING, and RIG-1, suppress the phosphorylation of TBK1 and IRF3, suppress nuclear translocation of IRF3	Luciferase assay, Western blotting, Immunofluorescence assays	HEK293T, HeLa cells	(51)
**M**	Suppress the phosphorylation of IRF3, TBK1, IKKα/β, p65	Luciferase assay, qRT-PCR, Western blotting	HEK293T	(13)
**IFN signaling inhibition**				
**NSP1**	Suppress STAT1 phosphorylation	Western blotting	HEK293T cells	(43)
**NSP6**	Suppress STAT1 and STAT2 phosphorylation	Western blotting	HEK293T cells	(43)
**NSP13**	Suppress STAT1 and STAT2 phosphorylation	Western blotting	HEK293T cells	(43)
**ORF3a**	Suppress STAT1 phosphorylation	Western blotting	HEK293T cells	(43)
**ORF6**	Inhibit STAT1 nuclear translocation	Immunofluorescence assays	HEK293T cells	(43, 48)
**ORF7a**	Suppress STAT2 phosphorylation	Western blotting	HEK293T cells	(43)
**ORF7b**	Suppress STAT1 and STAT2 phosphorylation	Western blotting	HEK293T cells	(43)
**ORF8**	Interact with MHC-I and mediate its down regulation	Western blotting	HEK293T cells	(52)
**ORF10**	Degrade MAVS	Western blotting	HEK293T cells	(53)

IRF3, Interferon regulatory factor 3; TBK1, TANK binding kinase 1; MDA5, melanoma differentiation-associated gene 5; MAVS, mitochondrial antiviral signaling; TRIF, TIR‐domain‐containing adapter‐inducing interferon‐β; STING, stimulator of IFN genes; RIG-1, retinoic acid-inducible gene I; STAT1, signal transducer and activator of transcription 1; STAT2, signal transducer and activator of transcription 2; MHC-I, Major histocompatibility complex I; IKKα/β, inhibitor of nuclear factor-κB (IκB) kinase alpha/beta.

## NSP6 inhibits IFN-β production by targeting IRF3 and IFN-I signaling by inhibiting STAT1/STAT2 phosphorylation

NSP6 inhibits IFN-β production and suppresses about 40% luciferase activity (43, 54). The distinct components of RIG-1 pathway were studied to identify which step of IFN-β production was inhibited by NSP6. The results showed that luciferase activity was suppressed when IFN-β promoter was induced by IKKϵ, TBK1, or MAVS, suggesting that NSP6 may inhibit IFN-β production by targeting IRF3 (before the activation of IRF3) or other component(s) upstream of IRF3 (that is between IRF3 and TBK1/IKKϵ) (43).

Similarly, the same study showed that NSP6 could modulate the phosphorylation of TBK1 and IRF3 (43). NSP6 overexpression followed by poly (I:C) transfection inhibited approximately 57% of IRF3 phosphorylation. However, no effect on phosphorylation of TBK1 was observed. Hence, it may be inferred that NSP6 binds TBK1, which decreases IRF3 phosphorylation, leading to a reduction of IFN-β production (**Figure 3**, **Table 1**) (43). Moreover, NSP6 also antagonizes IFN-I signaling by inhibiting the phosphorylation of STAT1 and STAT2 (**Figure 3**, **Table 1**). NSP6 could inhibit STAT1 phosphorylation by about 33%–46%, while STAT2 phosphorylation by about 33%–50% (43).

## NSP8 interacts with MDA5 to inhibit the phosphorylation of IRF3 and TBK1

NSP8 interacts with MDA5 and antagonizes the phosphorylation of IRF3 and TBK1. MDA5 is the most upstream sensor in the innate immune system and is involved in the recognition of dsRNA. NSP8 interacts with the CARD domain of MDA5 to downregulate the antiviral immune responses (44). Tertiary structures of NSP8, MDA5 CARD domain, and K63-Ub were determined, and simulation results showed that at the N terminal region of NSP8, there is a short α-helix that covers an area that interacts with K63-Ub. It is already known that the CARD domain of MDA5 undergoes k63-linked polyubiquitination and recruits MAVS to form a signalosome. The structural predictions of the interaction between NSP8 and MDA5 CARD domain showed that NSP8 may interrupt k63-linked polyubiquitination and MAVS recruitment, leading to the inhibition of MDA5 activation (55). Therefore, NSP8 inhibits K63-linked polyubiquitination of MDA5 by interfering with MDA5-MAVS signalosome.

Clinical data of COVID-19 patients revealed that disease severity is related to cytokine storm (56). NSP8 overexpression could downregulate the expression of cytokines, including IL-1β, IL-2, IL-5, IL-6, CCL-20, IFN-β, TNF-α, and ISGs IFIT1 and IFIT2 (44). These results indicate that NSP8 strongly impairs the expression of genes involved in antiviral immune and inflammatory responses.

NSP8 expression significantly inhibits the phosphorylation of TBK1, IRF3, IKKα/β, and p65 (**Figure 3**, **Table 1**). The NF-κB signaling was greatly inhibited, as delineated by decreased p65 phosphorylation. It also inhibits the activation of IRF3 and NF-κB. Moreover, NSP8-downregulated innate immune responses were dependent on MAVS, acting on either MAVS or upstream signals (44). These results suggest that NSP8 targets the upstream components of IFN-I signaling pathway.

## NSP12 inhibits IRF3 nuclear translocation by attenuating IFN-β production

NSP12 inhibits poly (I:C) and SeV-induced IFN-β promoter activation (45, 48). NSP12 overexpression inhibits IFN-β promoter activation triggered by MAVS, MDA5, RIG-IN, and IRF3-5D. NSP12 does not physically interact with IRF3. However, it decreases the nuclear translocation of IRF3 without impairing its phosphorylation (**Figure 3**, **Table 1**) (45). However, another study found that NSP12 is not an IFN-β antagonist (57). The induction of NSP12 does not affect the production of IFN-β both at mRNA and protein levels. The differences in results of these two studies may be due to different experimental setups. Therefore, cautions are required while interpreting SARS-CoV-2-related luciferase assays, as different tag proteins and backbones of plasmid could influence the results (57).

## NSP13 interacts with TBK1 and antagonizes IFN-I signaling by inhibiting STAT1 and STAT2 phosphorylation

NSP13 inhibits SeV-mediated promoter activity of NF-κB by about 2-fold. It also inhibits the activation and nuclear translocation of NF-κB as it reduces the levels of p-NF-κB when TBK1 and NSP13 co-overexpressed (46). NSP13 physically binds with TBK1, determined by Co-immunoprecipitation (Co-IP) experiments (14, 43, 46). In further experiments, it was found that IFN-β and ISRE promoter activities were downregulated after NSP13 overexpression induced by TBK1. This suggests that NSP13 antagonizes IFN response by suppressing IRF3 and TBK1 phosphorylation (**Figure 3**, **Table 1**) (46). Another study also suggests that NSP13 inhibits IFN-β production and suppresses about 48% luciferase activity (43). Furthermore, NSP13 targets IRF3 to inhibit IFN-β production by targeting IRF3 (before the activation of IRF3) or another protein upstream of IRF3 (between IRF3 and TBK1/IKKϵ) (43).

Furthermore, phosphorylation of TBK1 and IRF3 mediated by NSP13 was investigated (43). NSP13 overexpression followed by poly (I:C) treatment inhibited about 75% IRF3 phosphorylation. NSP13 could also inhibit the phosphorylation of TBK1 in a dose-dependent manner. Hence, it may be deduced that NSP13 interacts with TBK1 and inhibits its phosphorylation, leading to suppression of IRF3 activation and IFN-β production (**Figure 3**, **Table 1**) (43). NSP13 significantly suppresses STAT1/2 phosphorylation, suggesting its role in antagonizing IFN-I signaling. Moreover, NSP13 also inhibits the nuclear translocation of STAT1 during IFN-I signaling (**Figure 3**, **Table 1**) (43).

The recent mutations found in NSP13 make it a stronger IFN antagonist. The different mutations are P77L, Q88H, D260Y, E341D, and M429I, which were observed in different variants of SARS-CoV-2. Structural and biophysical analysis justified the stronger binding of these mutants with TBK1 and helped in more evasion from the host immune system (58). Therefore, it could be deduced that with the evolution of different variants, the capability of SARS-CoV-2 to evade the host immune system will increase.

## NSP14 inhibits IFN-β activation and IRF3 nuclear translocation

Among CoVs, NSP14 is highly conserved and exhibits approximately 99% amino acid similarity with its SARS-CoV counterpart (59). NSP14 has a guanine–N7-methyltransferase and 3’ to 5’ exoribonuclease activity (60). Mutations in the Zinc finger motif and the active site of the exonuclease domain result in a lethal phenotype of this virus (61).

In addition to NSP1, NSP14 also inhibits protein translation in cells (35, 59). The role of NSP14 in IFN-β and ISG production has been well documented (47, 48, 59). Overexpression of NSP14 suppresses the production of endogenous ISG proteins but does not affect their mRNA levels. Instead, inhibition of translation was shown to be responsible for the suppression of endogenous expression of ISGs by NSP14 (59).

In another study, NSP14 was found to inhibit SeV-mediated IFN-β activation. In addition, NSP14 was able to recapitulate this inhibition when IFN-β promoter activity was induced upon overexpression of RIG-I or MDA5 (48). NSP14 was also found to inhibit IFN production upon RIG-1 activation, and it significantly inhibited SeV-mediated nuclear translocation of IRF3 (**Figure 3**, **Table 1**) (47).

## NSP15 inhibits IFN production and IRF3 nuclear localization

NSP15 was shown to potentially suppress the production and signaling of IFN when the N-terminus RIG-1, an upstream activator of IFN signaling, was used as a potent inducer of IFN production (47). Furthermore, NSP15 significantly inhibits the nuclear localization of IRF3 upon SeV infection (**Figure 3**, **Table 1**) (47).

## ORF3a antagonizes IFN-I signaling by phosphorylating STAT1

Among all accessory proteins of SARS-CoV-2, ORF3a is the largest, with 275 amino acid residues. It shares approximately 72.7% similarity with the SARS-CoV ORF3a protein (62–64). ORF3a was shown to suppress more than 40% ISRE promoter activity and significantly suppress IFN-I signaling (43). It also suppresses STAT1 phosphorylation by 33%–46%; however, STAT2 phosphorylation was only marginally suppressed. Moreover, ORF3a suppresses STAT1 nuclear translocation during IFN-I signaling (**Figure 3**, **Table 1**) (43). Furthermore, ORF3a also induces lysosomal damage, necrotic cell death, and cytokine storms (65).

SARS-CoV-2 infection was shown to induce a pro-inflammatory cytokine response through cGAS-STING and NF-κB in human epithelial cells (66, 67). Inflammatory responses were observed in patients and could be therapeutically targeted to suppress severe disease symptoms. ORF3a has a unique ability to inhibit STING. ORF3a interacts with STING to inhibit NF-κB signaling by blocking the nuclear accumulation of p65. ORF3a therefore can antagonize immune activation induced by the cGAS-STING (67).

## ORF6 inhibits IRF3 nuclear translocation and hampers IFN-I signaling by blocking STAT1 nuclear translocation

SARS-CoV-2 ORF6, which contains 61 amino acid residues, shows low similarity to SARS-CoV ORF6 (64). All serbecoviruses, including SARS-CoV and SARS-CoV-2, encode this protein. However, no orthologs of this protein have been found in other β-CoVs, i.e., MERS-CoV, murine hepatitis virus (MHV), and OC43 (49). SARS-CoV ORF6 is known to counteract host antiviral responses at multiple steps of the innate immune pathway (68). ORF6 of SARS-CoV-2 localizes predominantly in the cytoplasm but can also be found in the Golgi apparatus and ER. ORF6 inhibits IFN-β promoter activation in a dose-dependent manner induced by either poly (I:C) or SeV (43, 48). Moreover, ORF6 inhibits IFN promoter activation mediated by MAVS, MDA5, RIG-I, and IRF3-5D. ORF6 inhibits IFN-β production at IRF3 levels or downstream of it. Furthermore, ORF6 overexpression inhibited SeV-induced nuclear translocation of IRF3. The amino acid residues 53–61 at the C-terminal tail of ORF6 were important for this antagonistic activity (48).

Furthermore, ORF6 overexpression does not affect IRF3 phosphorylation but significantly blocks its nuclear translocation (**Figure 3**, **Table 1**). Karyopherin α 1-6 (KPNA1-6) is responsible for the nuclear translocation of IRF3, IRF7, and STAT1 (69). ORF6 binds to KPNA2 but not to other KPNAs; therefore, it was suggested that ORF6 inhibits IFN-β production by interacting with KPNA2 and blocking IRF3 nuclear translocation (43). Inhibition of IRF3 nuclear translocation was also reported in another study, which suggests that residues E46 and Q56 are essential in providing ORF6 the antagonistic activity. Moreover, it was found that the C-terminal region of ORF6 was responsible for anti-innate immune activity by significantly inhibiting the nuclear translocation of IRF3 (49).

ORF6 also significantly suppresses IFN-I signaling (43, 48) and inhibits IFN-α- or IFN-β-induced ISRE and ISG56 promoter activities, suggesting that ORF6 antagonizes the downstream IFN signaling (43, 48). The overexpression ORF6 only marginally suppresses the phosphorylation of STAT1 and STAT2 (43). However, another study showed contradictory results that ORF6 does not suppress STAT1 phosphorylation (48). These observations indicate that ORF6 might suppress a step downstream of the STAT1/STAT2 phosphorylation (43). Moreover, ORF6 also suppresses nuclear translocation of STAT1 *via* ORF6/KPNA2 interaction, thereby inhibiting IFN-I signaling (**Figure 3**, **Table 1**) (43). ORF6 also inhibits IFN-β, ISRE, and NF-κB promoter activities in a dose-dependent manner. ORF6 overexpression inhibits the expression of the ISRE promoter, suggesting that different mechanisms are involved to regulate the IFN pathway. Furthermore, ORF6 suppresses SeV-induced mRNA levels of IFN-β, ISG56, and ISG54 (50).

ORF6 inhibits nuclear translocation of STAT1 and inhibits IFN signaling yet by a different mechanism as well. It interacts with nucleoporin 98 (Nup98)–ribonucleic acid export 1 (RAE1) (Nup98-RAE1) complex and antagonizes IFN signaling by inhibiting nuclear translocation of STAT1 (70) (**Figure 3**). The C-terminal region of ORF6 is important for this binding. The binding was impaired and IFN antagonist activity was abolished when Methionine (M) at position 58 was substituted with arginine (R) in ORF6 (71). ORF6 blocks STAT1 nuclear translocation by interacting with the Nup98-RAE1 complex and disrupts the interaction between Nup98 and importin-β1/importin-α1/PY STAT1 complex, thus preventing the binding of this complex at the nuclear pore. Moreover, SARS-CoV ORF6 binds to the Nup98-RAE1 complex, and ORF6s from both viruses share the same binding site on this complex (10). Nup98 is identified as a critical factor hijacked by SARS-CoV-2 to inhibit IFN signaling. Overexpression of Nup98 successfully rescues the ORF6-mediated inhibition of STAT1 nuclear translocation. Some other studies confirmed the interaction of ORF6 with the Nup98-RAE1 complex, suggesting that ORF6 specifically targets Nup98 to block STAT nuclear import (71–74).

The emergence of new variants of SARS-CoV-2 has enhanced its virulence and human-to-human transmission. The alpha (B.1.1.7) variant, which belongs to variants of concern (VOCs), shows enhanced suppression of innate immune responses in airway epithelial cells compared to first wave isolates (75). The alpha variants exhibit markedly increased levels of subgenomic RNA and protein levels of ORF6. The resulting increased levels of ORF6 protein inside host cells after infection increase the capability of ORF6 to antagonize the nuclear translocation of IRF3 and STAT1 proteins and hence antagonize IFN signaling more efficiently (75).

## ORF7a and ORF7b inhibits IFN-I signaling by suppressing STAT1 and/or STAT2 phosphorylation

ORF7a and ORF7b contain 121 and 43 amino acid residues (64). ORF7a was shown to inhibit STAT2 phosphorylation by approximately 33%–50% but only marginally suppress STAT1 phosphorylation (**Figure 3**, **Table 1**). ORF7b, on the other hand, suppressed STAT1 phosphorylation by 33%–46% and suppressed STAT2 phosphorylation by 33%–50%. Both ORF7a and ORF7b were shown to suppress ISRE promoter activity by approximately 40% and STAT1 nuclear translocation during IFN-I signaling (**Figure 3**, **Table 1**) (43).

## ORF8 inhibits IFN-I signaling pathway and downregulates MHC-I

ORF8 is the most puzzling gene of CoVs (76). It contains 121 amino acid residues with less than 20% sequence identity to SARS-CoV ORF8 (77). It contains a signal sequence for ER import. Antibodies to ORF8 are among the principal biomarkers of SARS-CoV-2 infection (78). Moreover, several studies have delineated the role of ORF8 in immune evasion (50, 78, 79). Since the emergence of SARS-CoV-2, several mutations in ORF8 have been recorded. These mutations, which include L84S (80, 81), V62L, S24L (82), and W45L (79), have been observed in different variants of SARS-CoV-2. Different mutants in ORF8 altered their binding efficiency to IRF3 such as W45L mutant was found to bind more stringently to IRF3, indicating its more profound role in immune evasion (79). Therefore, different variants will have different capabilities for evading the host immune system due to mutations in ORF8.

ORF8 has also been found to interact with major histocompatibility complex I (MHC-I) and mediate its downregulation (**Figure 3**, **Table 1**). Cells overexpressing MHC-I were found to be targeted for lysosomal degradation by autophagy. ORF8 impairs the activity of antigen-presenting cells; therefore, blocking ORF8 could be used to improve the immune system (52).

The role of ORF8 in IFN antagonism was also described in another study (50). Cells were cotransfected with NF-κB, IFN-β, or ISRE reporter plasmids and ORF8-overexpressing and respective control plasmids followed by SeV induction. ORF8 significantly inhibited the promoter activity of all three elements (NF-κB, IFN-β, and ISRE). In addition, it was found that ORF8 inhibits the expression of ISRE promoter, suggesting that it may adopt several mechanisms to regulate the host interferon pathway. Moreover, ORF8 significantly suppresses SeV-induced mRNA expression of IFN-β, ISG54, and ISG56 (50).

## ORF9b antagonizes IFN-I and IFN-III by targeting multiple signaling pathways

ORF9b encodes a protein of 97 amino acid residues and significantly inhibits the production of IFN-I by targeting mitochondria (53, 83). Antibodies against ORF9b were detected in convalescent sera from SARS-CoV and SARS-CoV-2 patients (84, 85). Therefore, the role of ORF9b concerning IFN-I production is obvious (86). To determine which host proteins interact with ORF9b, a biotin-streptavidin affinity purification mass spectrometry approach was used. Translocase of outer membrane 70 KDa Subunit (TOM70) was shown to bind most efficiently to ORF9b. Co-IP experiments further validated these findings. TOM70 is a mitochondrial import receptor that is important for MAVS activation of IRF3 and TBK1 (75). Furthermore, SARS-CoV ORF9b can also bind TOM70. Therefore, ORF9b and TOM70 binding are conserved in SARS-like CoVs. Two domains of TOM70 protein, i.e., the core and the C-terminal are important for this interaction. ORF9b localizes to the outer membrane of mitochondria as TOM70 is also localized at this site. The alpha variants have also markedly increased subgenomic RNA and protein levels of the ORF9b (75). ORF9b expression alone suppresses the innate immune response by binding to TOM70 (**Figure 3**). The binding of ORF9b and TOM70 was regulated by phosphorylation. Mutating ser53 alone or both ser50 and ser53 in ORF9b to phosphomimetic glutamic acid interrupted the binding between ORF9b and TOM70. Therefore, unphosphorylated ORF9b is highly active soon after virus infection to allow effective innate antagonism of the host (75).

Mitochondria and TOM70 play important roles in the IFN-I responses (87). ORF9b significantly inhibits IFN-β production. Moreover, TOM70 overexpression was shown to largely rescue IFN-β production from ORF9b-mediated inhibition. Therefore, therapeutic agents that inhibit the binding of ORF9b and TOM70 in COVID-19 patients could be developed (86).

Type I and type III IFN responses are inhibited by ORF9b through multiple antiviral pathways (51). Cells overexpressing ORF9b, followed by poly (I:C) or SeV induction, downregulate the expression of IFN-β, IFN-λ1, ISG56, and CXCL10. To map the step at which ORF9b could exert its inhibitory effects, it was found that ORF9b could antagonize the activities of IFN-β-Luc, IFN-λ1, ISRE-Luc reporters induced by MAVS, MDA5, TBK1, RIG-IN, but not those induced by IRF3/5D. These results suggest that ORF9b inhibits IFN production at a step upstream of IRF3. ORF9b appeared strongly localized in mitochondria but weakly colocalized with ER and Golgi. Moreover, ORF9b does not interact with IRF3 but it does interact with MDA5, MAVS, TRIF, TBK1, STING, and RIG-1. Therefore, ORF9b targets multiple components of the innate system to inhibit IFN production. Furthermore, it was found that ORF9b suppresses the phosphorylation of TBK1. This phosphorylation suppression is induced by all three important antiviral pathways, i.e., cGAS-STING, TLR3-TRIF, and RIG-I/MDA5-MAVS. ORF9b also suppresses SeV-induced IRF3 nuclear translocation and phosphorylation (51).

## ORF10 suppresses IFN-I signaling by degrading MAVS

The ORF10 protein contains 38 amino acid residues, but the sequence of SARS-CoV-2 ORF10 is different from ORF10s of other CoVs (64). Since the recent pandemic began, no specific function was attributed to this protein; however, a recent study described its role in the suppression of IFN-I signaling (53). ORF10 significantly antagonizes IFN-I and ISG expression and degrades MAVS *via* mitophagy by accumulating LC3 inside mitochondria. ORF10 translocates to mitochondria and induces mitophagy by interacting with Nip3-like protein X (NIX) and LC3B. IFN-I signaling inhibition is blocked when NIX is knocked down. Therefore, ORF10 suppresses the IFN signaling pathway by inhibiting MAVS expression and promoting viral replication (**Figure 3**, **Table 1**) (53, 88).

## Membrane (M) protein interacts with MAVS and inhibits the phosphorylation of TBK1 and IRF3

Membrane (M) is a glycosylated structural protein consisting of 222 amino acid residues. The N-terminal part of this protein contains three membrane spanning domains that are responsible for the assembly of viral particles (13, 14).

M protein inhibits the activation of IFN-β promoter, ISRE, and NF-κB in a dose-dependent manner induced by SeV (13). Furthermore, stable cell lines expressing M protein inhibit SeV- or poly (I:C)-induced IFNB1, ISG56, CXCL10, and TNF transcription. Stable expression of ACE2 in HEK293 cells (HEK293-ACE2) suppressed SARS-CoV-2-induced transcription of IFN-β1 and downstream antiviral when overexpressed with M protein. In addition, this protein inhibits IRF3, TBK1, IKKα/β, and p65 phosphorylation in cells (13).

M inhibits ISRE and IFN-β promoter activities mediated by MAVS, RIG-I-CARD, and MDA5 overexpression but not by TBK1. M suppresses NF-κB expression, which is mediated by MDA5, MAVS, and RIG-I-CARD but not by p65. Therefore, it could be deduced that M inhibits innate antiviral signaling at the MAVS level. M protein physically interacts with MAVS (at its transmembrane domain) but not with MDA5, TBK1, or RIG-I. Moreover, M protein disturbs the recruitment of IRF3, TRAF3, and TBK1 to the MAVS complex, and this impairment inhibits the innate antiviral response (13).

## Other proteins

In addition to the abovementioned proteins, the following proteins are also found to play a role in IFN antagonism. However, their detailed mechanisms require further investigations.

NSP5 is a protease that cleaves ORF1a and ORF1b into peptides and blocks MAVS-induced IFN-β production. The C145A mutant of NSP5 abrogates the proteolytic activity and fails to inhibit the activation of IFN-β. Therefore, the proteolytic activity is indispensable for NSP5 to suppress IFN-β production (54).

ORF3b, which contains 22 amino acid residues, is considerably shorter than SARS-CoV ORF3b (153 amino acid residues). The results of a luciferase reporter assay suggested that SeV-induced promoter activity of IFN-β was suppressed after overexpression of ORF3b (89).

ORF9c contains 73 amino acid residues and shares 74% sequence identity with SARS-CoV ORF14 and approximately 94% sequence identity with bat SARS-CoV ORF14. It has a putative transmembrane domain that interacts with M protein in various cellular compartments. This interaction disturbs the antiviral process in lung epithelial cells. The expression of this highly unstable protein disturbed IFN signaling, complement signaling, and antigen presentation and induced IL-6 signaling. ORF9c enables evasion of the immune system and coordinates cellular changes that are important in the life cycle of SARS-CoV-2 (90).

Nucleocapsid (N) protein inhibits the promoter activities of IFN-β, ISRE, and NF-κB. N protein also suppresses SeV-induced IFN-β, ISG54, and ISG56 mRNA expression levels but does not inhibit the expression from the ISRE promoter (50). The alpha variants have also markedly increased subgenomic RNA and protein levels of N protein (75). Therefore, the already IFN antagonistic activity of N against IFN could be highly enhanced in alpha variants.

## Perspectives

Innate and adaptive immune responses are considered fundamental elements of host defense against viral infections, but SARS-CoV-2 has devised strategies to evade the immune system. In this article, we summarized the roles and mechanisms of actions of different SARS-CoV-2 proteins playing important roles in the host’s immune evasion. SARS-CoV-2 proteins inhibit the production and signaling of IFNs by different mechanisms. For instance, NSP12, NSP14, NSP15, ORF6, and ORF9b inhibit IRF3 nuclear translocation (43, 45, 47, 48, 50, 51), whereas NSP1, NSP6, NSP8, NSP13, ORF3a, ORF7a/b, ORF9b, and Membrane proteins are involved in the phosphorylation inhibition of various components of the immune system (13, 43, 44, 46, 51). ORF8 downregulates MHC-I to evade the host immune system (52). NSP8 interacts with MDA5 to impair its K63-linked polyubiquitination and mediate immune evasion (44). ORF9c interacts with membrane proteins and impairs the antiviral process in lung epithelial cells (90). These examples demonstrate that each protein of SARS-CoV-2 may perform multiple functions and mediate immune evasion by different mechanisms. Moreover, mutations in the viral genome also play important roles in more aggressive infections. Different variants of interest (VOIs) and VOCs are evolved that favor enhanced human-to-human transmission. Therefore, it is necessary to study host immune evasion in more variants, so that potential therapeutic strategies can be developed. In addition, the inhibitory potential of antagonizing proteins may be different in different experimental setups. Further investigations are required to gain more insights and information about immune responses and COVID-19 interactions. Detailed mechanistic studies of NSP5, ORF3b, ORF9c, and N proteins will further elucidate the pathogenesis of SARS-CoV-2 and therefore need further investigations.

## Author contributions

ZX presented the concept and edited the article. FR presented the concept and wrote the article. FR, MS and SK designed the figures. FR, AS and SL edited and review the article. All authors contributed to the article and approved the submitted version.

## Funding

This work was supported by the Guangxi BaGui Scholars Program Foundation (2019A50).

## Conflict of interest

The authors declare that the research was conducted in the absence of any commercial or financial relationships that could be construed as a potential conflict of interest.

## Publisher’s note

All claims expressed in this article are solely those of the authors and do not necessarily represent those of their affiliated organizations, or those of the publisher, the editors and the reviewers. Any product that may be evaluated in this article, or claim that may be made by its manufacturer, is not guaranteed or endorsed by the publisher.
